# Electrophysiological Correlates of Encoding Indicate Altered Processing but Comparable Subsequent Memory Effects Across Virtual Reality and Desktop‐Based Conditions

**DOI:** 10.1111/ejn.70664

**Published:** 2026-08-27

**Authors:** Joanna Kisker, Merle Sagehorn, Thomas Gruber

**Affiliations:** ^1^ Experimental Psychology I, Institute of Psychology Osnabrück University Osnabrück Germany

**Keywords:** encoding, subsequent memory, theta‐band response, virtual reality

## Abstract

Successful episodic memory formation emerges from the interplay of perceptual, attentional, contextual, and elaborative processes. The relative contribution of these processes to encoding may vary with the conditions under which information is encountered. In particular, different presentation modalities, like virtual reality (VR) and desktop‐based presentations (PC), can convey the same semantic identity of an item while differing in perceptual, spatial, and contextual information. Although VR‐ and PC‐encoded memories have been observed to differ in retrieval processes, it remains unclear whether these differences arise during encoding. The present study addressed this question by comparing successful object encoding across presentation modes. Participants incidentally encoded everyday objects in VR and on a PC, and successful encoding was assessed by relating encoding‐related EEG activity to later memory. Subsequently remembered objects elicited reliable ERP subsequent memory effects, linked to semantic, contextual, and elaborative encoding processes, as well as an enhanced theta‐band response, indicating contextual integration. However, these effects were not modulated by presentation mode, consistent with comparable memory performance across conditions. In contrast, presentation mode affected encoding activity irrespective of subsequent memory success: VR elicited a more negative early ERP and stronger theta‐band activity at frontal sensors than PC‐based presentation, potentially reflecting altered object evaluation and enhanced visuospatial integration. These modality‐dependent differences could not be attributed primarily to early visual or attentional processing, as they were not accompanied by differences in P1 or alpha‐band responses. Thus, VR altered general object processing during encoding, whereas the neural processes predicting later recognition were comparable across presentation modes.

## Graphical abstract

The study examined whether successful object encoding relies on comparable or modality‐specific neural processes across immersive virtual reality (VR) and desktop (PC) presentation. Encoding‐related EEG activity was analyzed in a subsequent memory paradigm using ERP and oscillatory markers. The findings suggest that successful memory formation relies on similar encoding‐related neural processes in immersive VR and conventional PC settings: Encoding in immersive virtual reality altered general electrophysiological processing, whereas subsequent memory effects were comparable across presentation modes.

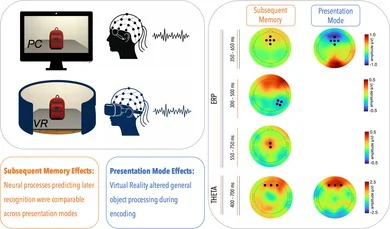

**Abbreviations**
CS: correct source retrieved
EEG: electroencephalography
EOG: electrooculogram
ERP: event‐related potential
HMD: head‐mounted display
ITI: inter‐trial‐intervall
PC: personal computer
SME: subsequent memory effect
VR: virtual reality

## 1 Introduction

The formation of memories depends on the neural and cognitive operations engaged, i.a., at the moment of encoding. Rather than reflecting a unitary mechanism, successful encoding emerges from the interplay of multiple processes, including visual perception, attention allocation, contextual integration, and semantic and elaborative processing (Kamp et al. 2017; Mecklinger and Kamp 2023; Paller and Wagner 2002). The relative contributions of these processes may thus depend not only on *what* is encoded but also on *how* information is encountered during encoding. Everyday objects, for instance, may be encoded differently when presented as pictorial stimuli than when experienced as three‐dimensional objects. Although the semantic identity of an object is similar across both formats, the perceptual, spatial, and contextual information available during encoding can differ substantially (Kisker, Johnsdorf, et al. 2025a; Romero and Snow 2019; Snow and Culham 2021) and add complexity to the stimulus (Nejati 2021). An especially informative comparison of these characteristics is between conventional desktop‐based (PC) and virtual reality (VR) head‐mounted display (HMD) conditions. Beyond 2D pictorial stimuli, immersive VR provides stereoscopic depth cues, egocentric perspective, and spatial context. Due to these immersive features, investigations of cognitive processes are increasingly conducted in VR to more closely mirror real‐life conditions (Johnsdorf et al. 2026; Kisker, Johnsdorf, et al. 2025a, 2025b; Schöne et al. 2023), since these features may shift the balance of the processes contributing to successful memory formation, depending on the mode of presentation.

Whereas the fundamental processes underlying retrieval remain consistent across different modalities, their relative contribution to successful retrieval can differ substantially (Johnsdorf et al. 2026; Kisker et al. 2021a, 2026; Kisker, Soethe, et al. 2025; Sagehorn, Kisker, et al. 2026). In a behavioral study, VR experiences were more strongly associated with vivid retrieval of contextual details of previously encountered objects, known as recollection, whereas PC experiences were more strongly associated with a relatively undifferentiated sense that an item had been encountered before, known as familiarity (Kisker et al. 2021a).

Beyond such behavioral outcomes, a growing number of studies have demonstrated that established electrophysiological markers can be reliably acquired in immersive VR despite the technical challenges associated with combining EEG and HMDs. Across different mnemonic domains, established ERP and oscillatory correlates have been successfully accessed in immersive VR, including those linked to implicit memory (Johnsdorf et al. 2023; Kisker et al. 2024), recognition‐memory (Johnsdorf et al. 2026; Kisker et al. 2026; Kisker, Soethe, et al. 2025), and visual working‐memory (Klotzsche et al. 2023; for a review on joint VR‐EEG studies, see Choi et al. 2023). Together, these findings indicate that immersive VR provides a valid platform for investigating memory‐related electrophysiological processes under more naturalistic conditions. Even more importantly, however, these studies indicate that VR can affect the retrieval dynamics and qualitative characteristics of later recognition (Kisker et al. 2026; Kisker, Soethe, et al. 2025). However, it remains unresolved whether such modality‐dependent differences ultimately reflect processes engaged during encoding that modulate consolidation as a downstream consequence, or arise primarily during later retrieval.

If modality‐dependent differences in retrieval processes originate from encoding, they are likely to reflect differences in the perceptual, attentional, contextual, or elaborative operations engaged when stimuli are first encountered. At the perceptual level, previous studies suggest largely comparable early visual processing across presentation modes, although early ERP amplitudes can differ in magnitude, presumably reflecting the additional depth and spatial cues provided by VR (Kisker, Johnsdorf, et al. 2025a). Likewise, theta‐band activity suggests enhanced visuospatial processing in VR, whereas evidence regarding attentional processing remains mixed and appears to depend on task demands (Kisker, Johnsdorf, et al. 2025b). On the one hand, several studies have reported comparable attentional processing of stimuli in VR and on a 2D desktop, as reflected in alpha‐band responses (Johnsdorf et al. 2026; Kisker, Johnsdorf, et al. 2025a; Sagehorn, Kisker, et al. 2026). However, others reported increased early attentional processes in VR during working memory tasks (Aksoy et al. 2021; Sagehorn, Johnsdorf, et al. 2026; Schubring et al. 2020).

Beyond perceptual‐attentional processing, immersive VR may create conditions that facilitate the contextual integration of encoded stimuli. Spatially coherent VR environments provide additional contextual information that can be integrated with object representations and may encourage the formation of richer associative memory traces (Parsons 2015; Slater and Wilbur 1997; Wrzus et al. 2024). Such item‐context integration is considered fundamental to episodic memory because successful remembering depends on binding an item to its spatial, temporal, and situational context (Diana et al. 2007; Eichenbaum et al. 2007; Johnson et al. 1993; Yonelinas et al. 2019). By embedding objects within a richer experiential context, VR can offer more cues for forming associations, situational meaning, or action‐related interpretations, which are linked through elaborative encoding (Kamp et al. 2017; Mecklinger and Kamp 2023; Paller and Wagner 2002). Accordingly, immersive VR may support contextual integration and elaborative encoding, potentially contributing to successful memory formation to a different extent than in conventional PC‐based experiences.

Taken together, previous findings provide evidence that the mode of presentation can modulate perceptual and attentional aspects of object processing, whereas effects on contextual integration and elaborative encoding are more strongly implied by the affordances of immersive VR. Initial electrophysiological evidence further suggests modality‐dependent differences during early processing stages related to memory formation (Johnsdorf et al. 2023). However, it remains unclear whether these differences, or the contextual and elaborative affordances of immersive VR more generally, predict successful memory formation rather than reflecting differences in initial object processing. Consequently, the current study focuses on whether memory formation under immersive VR and PC conditions relies on comparable or modality‐specific configurations of perceptual‐attentional, contextual, and elaborative encoding processes.

This question can be addressed by subsequent memory paradigms, in which neural activity elicited during encoding is analyzed in relation to subsequent retrieval, i.e., whether items are later remembered or forgotten (Paller and Wagner 2002; Rugg et al. 2002). Usually, subsequently remembered stimuli exhibit a more positive response during encoding than subsequently forgotten stimuli, resulting in a subsequent memory effect (SME). These SMEs thus provide a means of understanding the cognitive and neural processes underlying successful memory formation, rather than merely highlighting general differences between experimental conditions (i.e., between the two modes of presentation).

Successful and associative encoding have been linked to the frontal theta‐band response, which is also related to working memory, cognitive control, and episodic memory processes (Gruber et al. 2008; Hanslmayr et al. 2011; Hanslmayr and Staudigl 2014; Hsieh and Ranganath 2014; Nyhus and Curran 2010). During encoding, increases in the theta‐band response have been associated with long‐term memory formation and item‐context binding (Hanslmayr et al. 2011; Hanslmayr and Staudigl 2014). This oscillatory activity is thus expected to be especially sensitive to the formation of contextual relations across encoding and retrieval, rather than to successful retrieval alone. It is therefore linked to the extent to which successful encoding relies on controlled integration of a stimulus into contextual information.

Complementary to oscillatory measures, event‐related potential (ERP) SMEs provide temporally resolved markers of successful encoding, with frontal and parietal effects in the several‐hundred‐millisecond range associated with attentional, semantic, and elaborative processes that support later retrieval (Friedman and Johnson 2000; Mecklinger and Kamp 2023; Paller and Wagner 2002; Rugg et al. 2002). As emphasized by Mecklinger and Kamp (2023), ERP SMEs constitute a family of effects that differ in functional significance depending on their timing and topographical distribution. These effects have been linked to semantic processing (frontally distributed SME, approx. 300–500 ms), attention allocation and item‐context binding (parietally distributed SME, approx. 350–500 ms), and conceptual or associative processing (frontally distributed SME, sustained from 550 ms onward). Combining ERP and theta‐band response SMEs thus allows different temporal and functional aspects of successful encoding to be compared between modes of presentation, i.e., VR and PC.

In summary, the present study examined whether successful encoding of everyday objects relies on comparable or modality‐specific neural processes in immersive VR and a matched PC condition. To this end, theta‐band and ERP SMEs were compared for subsequently remembered and forgotten objects across both presentation modes to determine whether perceptual‐attentional, contextual, and elaborative encoding processes differed between modalities. Based on previous findings indicating modality‐specific differences in visual processing, memory performance, and retrieval mechanisms (Johnsdorf et al. 2023, 2025, 2026; Kisker et al. 2021b, 2021a, 2026; Kisker, Soethe, et al. 2025), we expected that VR and PC differ in the degree to which specific encoding‐related processes predict later memory. In particular, if successful encoding in VR relies more strongly on spatial and contextual integration, modality‐dependent SMEs should be evident in frontal theta‐band activity. By specifying the subprocesses contributing to encoding, ERP SMEs were expected to reveal whether semantic, contextual, and elaborative subprocesses contribute similarly or differently to successful encoding across VR and PC. Given that the size‐judgment task required access to semantic object knowledge in both conditions, semantic SMEs were expected to be largely comparable, whereas contextual, attentional, and elaborative SMEs might differ if VR‐specific affordances facilitate successful encoding.

## 2 Methods

### 2.1 Sample Size and Participants

The study procedures were approved by the ethics committee of Osnabrück University (Ethik‐59/2025). Participants gave informed written consent and were unaware of the research question and hypotheses. They were compensated with partial course credits or with monetary compensation. G* Power (Faul et al. 2007) was used for an a priori power analysis. The analysis focused on a 2 × 2 repeated‐measures ANOVA (rmANOVA) with an estimated effect size of *f =* 0.40. The estimated effect size was derived from two previous studies comparing VR and PC conditions during initial stimulus presentation (Johnsdorf et al. 2023; Kisker et al. 2024), as well as effect sizes in remember/forgotten effects (Kamp et al. 2017, 2025). The power analysis indicated a minimum sample size of 15 participants. To account for potential technical issues and the power analysis in a forthcoming companion paper (focusing on retrieval processes), a target of 30 datasets was established. Ultimately, 42 participants were recruited from Osnabrück University and screened for psychological or neurological issues. Four participants withdrew before data collection, and two participants were excluded due to unacceptable data quality, resulting in 36 participants (*M*_age_ = 22.25; SD_age_ = 3.68; 94.4% right‐handed; sex: 63.9% female, 36.1% male). Among these, 58.3% had prior experience with head‐mounted displays (HMD) but were not regular users. 50.0% used computers regularly for gaming or entertainment.

### 2.2 Stimulus Material

**FIGURE 1:**
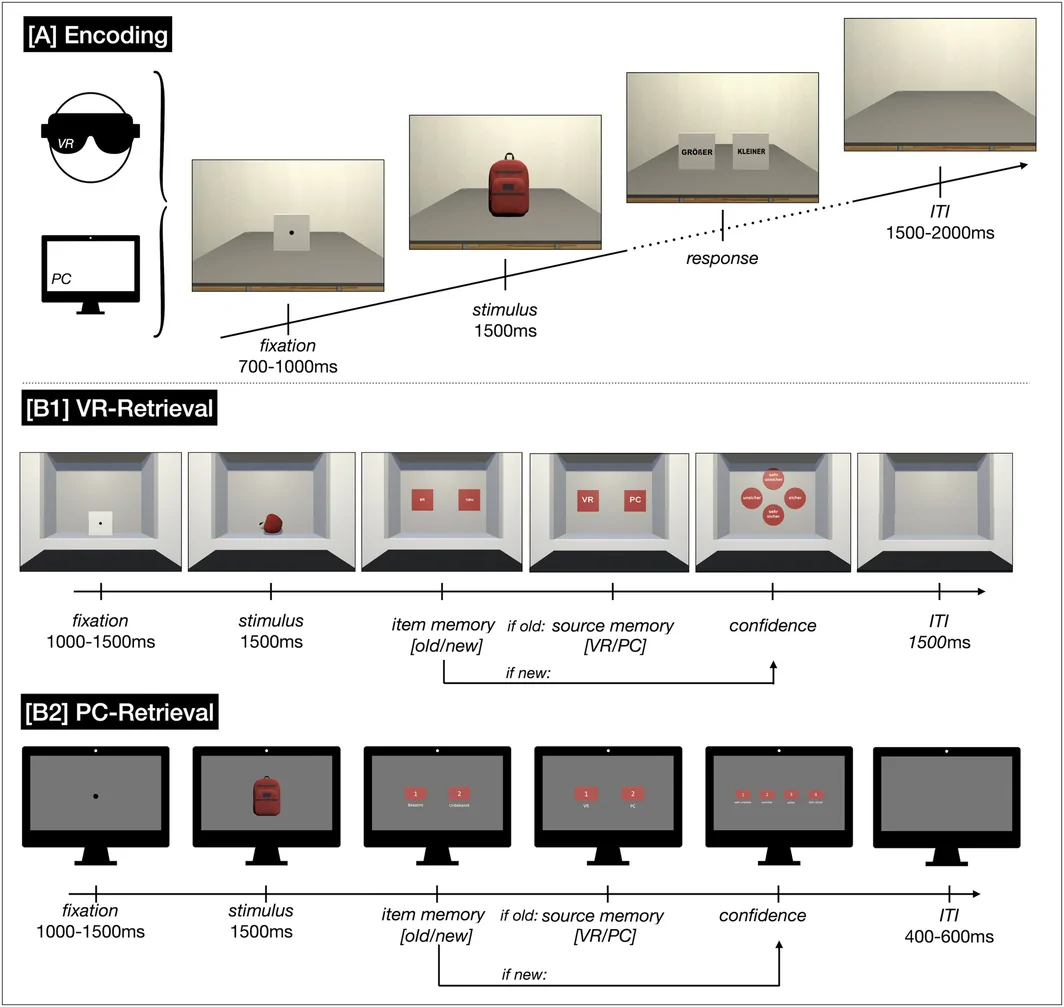
Schematic representation of the experimental procedure. *Note.* [A] Participants encoded 200 stimuli each in VR and on a PC in a within‐subjects design. The encoding phase took approximately 20 min per condition, including two automatic breaks after 70 and 135 trials, respectively. Participants were asked to compare the estimated real‐life size of each object with that of a conventional shoebox. [B] The retrieval task took approximately 45 min per condition [B1: VR condition, B2: PC condition], including two self‐paced breaks after 100 and 200 trials, respectively. If participants rated an item as old, a source‐identification task was administered to assess retrieval of the presentation mode (VR or PC). ITI = Inter‐trial‐interval.

The stimuli consisted of 640 3D objects. The objects were generated using the AI tool Tripo3D. The objects were derived from a word list by Birchenough et al. (2017) and filtered to include specific, depictable objects only. The objects were pre‐categorized by their real‐life size: 311 were small enough to fit in a shoebox at life size (e.g., a tennis ball, a smartphone), and 329 were too large to fit in a shoebox (e.g., a shelf, a cow; see behavioral task and Figure 1). This categorization was established for the incidental learning task (see 2.3: Procedure). Seven additional objects from a distinct database (Tromp et al. 2020) were included for training trials to prevent overlaps with the experimental trials.

### 2.3 Procedure

The experiment comprises an encoding phase and a retrieval phase. The publication at hand focuses on the encoding phase of the experiment, whereas the analysis of the retrieval phase will be addressed in a forthcoming companion paper.

The study follows up on two prior studies from our working unit and addresses questions raised in those studies (Kisker et al. 2026; Kisker, Soethe, et al. 2025). The methodology is therefore strongly based on these studies. All studies adhered to established source memory designs, beginning with an incidental encoding phase, followed by an unannounced recognition memory test, in a within‐subjects design. EEG data were obtained during both phases of the experiment using a 64‐channel EEG system.

#### 2.3.1 Encoding Phase

The encoding phase comprised two conditions: VR and PC. Both conditions were presented in a counterbalanced order and were developed using Unity 5 (version 2023.2.1f1; Unity Technologies, San Francisco, USA). The virtual camera's field of view was set to the default value of 60° in both conditions.

The VR condition was presented using an HTC Vive Pro 2 head‐mounted display, providing a per‐eye resolution of 2448 × 2448 pixels and a refresh rate of 90 Hz. Participants were positioned seated at a grey virtual table, 90 cm from the stimulus presentation location. The PC condition was presented using a 55″ display positioned 70 cm from the participant, offering a resolution of 1920 × 1080 pixels and a refresh rate of 60 Hz. The same virtual environment, including the table, was rendered in 2D for this condition. The visual angle was aligned across conditions using the size of the fixation cube as a reference (see Figure 1). The cube was displayed on the desktop in the PC condition, measuring 15 cm × 15 cm, and in the VR condition, measuring 19 cm × 19 cm. For each condition, the distance between the participants and the cube was adjusted to achieve a viewing angle of 12°. All stimuli were scaled to be maximized within the boundaries of the fixation cube. The viewing angle was calculated per object and participant by tracking the distance between the headset position and the stimulus. Averaged across trials and participants, this resulted in an average viewing angle of 13.35°.

Each condition included five training trials and 200 experimental trials. For each participant, 600 stimuli from the databases were randomly divided into three subsets: 200 VR encoding trials, 200 PC encoding trials, and 200 distractor items for the recognition memory task. Within each subset, 100 objects were larger, and 100 were smaller than a standard shoebox, based on real‐life size estimates.

Before the experimental trials, participants were verbally instructed, and any task‐related uncertainties were addressed. To mitigate potential fatigue and muscular strain, a two‐minute break was automatically scheduled after 70 and 135 experimental trials per condition, respectively. Each trial started with a fixation (700–1000 ms), followed by stimulus presentation (1500 ms). Subsequently, participants were cued to indicate whether the presented object's real‐life size was larger or smaller than a conventional shoebox, followed by an inter‐trial‐interval (ITI, 1500–2000 ms; see Figure 1). Responses were given by pressing the shoulder buttons on a standard gamepad. The training initiation, the start of the experimental trial, and the break offset were signaled visually by a green cube displaying a start symbol. Training completion, break onset, and the experiment's end were indicated by a red cube depicting a pause symbol. Each condition required approximately 20 min to complete, including the scheduled breaks.

#### 2.3.2 Delay

Upon completion of both encoding conditions, participants were relocated to the electroencephalogram (EEG) laboratory. To minimize potential confounding effects of rehearsal on subsequent recognition, participants completed a Sudoku puzzle. Participants continued working on the Sudoku until either successful completion or a 10‐min time limit was reached, with the earlier event serving as the cutoff criterion. Participants spent on average 8.2 (±2.20 SD) min on the Sudoku.

#### 2.3.3 Retrieval Phase

Each participant completed an unannounced recognition test in VR and PC conditions in a counterbalanced order. Per retrieval condition, 100 objects encoded in VR, 100 objects encoded on the PC, and 100 distractors were presented. Each subset included 50 objects smaller and 50 objects larger than a shoebox in real life. For the PC condition, the unannounced recognition test used 2D images of the 3D objects as retrieval cues. All stimuli were presented on a standard 24‐in. monitor (1020 × 1200 pixels) against a grey background matching the color of the encoding table. The participants viewed the stimuli at horizontal and vertical angles of 7.96° and 3.49°, respectively. For the VR condition, participants were seated in front of a virtual wall with a shelf‐like recess in which the objects were presented in 3D (see Figure 1). As in the preceding study, the environment did not visually replicate the encoding environment. This condition used the 3D models of the objects as retrieval cues. The viewing angle was calculated per object and participant by tracking the distance between the headset position and the stimulus. The viewing angle was calculated for each stimulus at its onset and averaged across trials and participants, yielding an average of 11.84°.

Following the task instruction, seven training trials were conducted, and participants were given the opportunity to clarify any remaining uncertainties before the experimental trials began. Each trial of the recognition task began with a 1000–1500 ms fixation period, followed by 1500 ms of stimulus presentation. Upon stimulus offset, a series of rating scales was presented sequentially (see Figure 1, Retrieval). Participants initially indicated stimulus recognition via an old/new judgment (item memory). For stimuli identified as “old,” a subsequent source identification task was administered. Participants indicated whether they encoded the item in the VR or PC condition (source memory). Stimuli categorized as “new” bypassed the source identification task. In both cases, participants rated their confidence in their response on a 4‐point Likert scale (1 = *very unsure*, 2 = *unsure*, 3 = *sure*, 4 = *very sure*). The entire recognition task required approximately 45 min per condition. To mitigate fatigue, participants were offered a break after every 100 trials, as well as a break between retrieval conditions. Breaks following 100 trials averaged 61 s in VR and 43 s in PC; and breaks after 200 trials averaged 65 s in VR and 56 s in PC, respectively. Upon completion of each condition, participants were asked to rate the subjective difficulty of the recognition task (0 = *extremely easy*, 10 = *extremely difficult*). After both conditions were completed, the EEG equipment was removed, and participants were debriefed.

### 2.4 Electrophysiological Recordings and Preprocessing

An EEG was recorded with a mobile 64‐channel active EEG system (LiveAmp, Brain Products, Gilching, Germany). Electrodes were placed according to the international 10–20 system. FCz served as the online reference, and an additional ground electrode was placed at FPz. During data collection, an online bandpass filter of 0.016–250 Hz was used. To ensure a good signal‐to‐noise ratio, electrode impedance was maintained below 10 kΩ, except for three participants, for whom it was below 15 kΩ. The live signal was checked for line noise and slow drifts, which were corrected before starting the paradigm. Data were recorded at 500 Hz sampling rate. Triggers indicating stimulus onset and participant responses were sent from Unity (VR condition) or Matlab (PC condition) via LSL (by SCCN; https://github.com/sccn/labstreaminglayer) to synchronize EEG and event triggers. Event timing was counterchecked and corrected using a photodiode.

#### 2.4.1 Preprocessing

Preprocessing was performed in MATLAB (R2024a, MathWorks) using EEGLAB functions (version 2024, Delorme and Makeig 2004). Due to massive muscle artifacts at electrodes FT9 and FT10 in 80% of the participants, these electrodes were excluded from all datasets prior to any preprocessing. The former reference channel FCz was reinstated as an additional data channel. The raw continuous data were resampled to 512 Hz, and each electrode was separately detrended before a FIR band‐pass filter (EEGLab function) was applied (high‐pass: 0.25 Hz, low‐pass: 30 Hz). Noise in the range of 1.24 Hz was removed using the *Zapline Plus* plugin (de Cheveigné 2020; Klug and Kloosterman 2022). Bad channels were identified using *Artifact Subspace Reconstruction* (ASR; default settings; Mullen et al. 2015). The identified channels were interpolated (VR: 5.97 ± 2.52 SD; PC: 5.75 ± 1.76 SD). The data were segmented into epochs from −500 to 1500 ms relative to stimulus onset. A baseline correction from −300 ms to stimulus onset was applied. Epochs containing blinks were detected and removed using statistical control of artifacts in dense array studies (*SCADS*; Junghöfer et al. 2000). On average, 36 trials were excluded per participant and condition due to blinks (VR: 36.75 ± 10.06; PC: 35.53 ± 10.42). An independent component analysis (ICA) was performed, and the *ICLabel* function (Pion‐Tonachini et al. 2019) was used to identify and remove artifacts labeled as eye (probability > 0.70), muscle, heart, line, and channel noise, and other (probability > 0.9). After excluding the respective components (VR: 8.58 ± 3.65; PC: 8.11 ± 4.21), the data were re‐referenced to the average reference (please see limitations and Data S1).

Trials were categorized by encoding condition (VR, PC) and by subsequent retrieval (remembered, forgotten), but, obviously, regardless of whether they were recalled in VR or on a PC. Trials were split into separate files containing PC‐encoded objects subsequently correctly categorized as old (PC remembered; 124.6 ± 17.7 trials), PC‐encoded objects subsequently falsely categorized as new (PC forgotten; 39.8 ± 17.2 trials), VR‐encoded objects subsequently correctly categorized as old (VR remembered; 125.3 ± 21.3 trials), and VR‐encoded objects subsequently falsely categorized as new (VR forgotten; 37.9 ± 17.4 trials), respectively. Depending on participants' responses, the number of trials per condition varied.

#### 2.4.2 Event‐Related Potentials

ERP analyses followed a literature‐guided, component‐specific approach. Based on the comprehensive review by Mecklinger and Kamp (2023), time windows and regions of interest (ROIs) were defined for a family of established ERP subsequent‐memory effects (SMEs). These effects include two frontally distributed effects with onsets around 300 and 550 ms, respectively, and a parietally distributed effect with an onset around 300 ms. Comparable effects have also been observed over central scalp regions and have been interpreted as reflecting a P300‐like SME (see e.g., Forester and Kamp 2023; Höltje et al. 2019). Based on the root mean square across frontal electrodes, the time windows of interest were set to 350–650 ms and 550–750 ms after stimulus onset for the frontal effects, and to 300–500 ms for the parietal effect. Electrode clusters were defined within the literature‐based ROIs by identifying the local maximum (or minimum, depending on component polarity) within each ROI and averaging across immediately adjacent electrodes within the same maximum or minimum, respectively. This procedure ensured that each electrode cluster captured the regional peak within the ROI while providing a more robust estimate than single‐channel measurements. Consequently, the frontal electrodes Fz, FCz, AFz, F1, and F2 were averaged for the time window from 350 to 650 ms, the frontocentral electrodes FCz and Cz were averaged for the time window from 550 to 750 ms, and the partietal electrodes P4, P6, CP4, and CP6 were averaged for the time window from 300 to 500 ms. This approach was chosen because previous SME research provides well‐established expectations regarding the temporal and spatial characteristics of the SMEs in the ERP. ROI‐based analyses are suited for testing specific hypotheses concerning established ERP components and facilitate direct comparison with previous SME studies. In contrast, mass‐univariate and cluster‐based permutation approaches would be more advantageous when little knowledge exists about the latency or scalp distribution of effects (Groppe et al. 2011).

To control for potential modality‐specific variations in early visual processing (e.g., depending on perceived stimulus size), the P1 was determined and included in the analyses. To this end, the electrodes Oz, O1, O2, POz, P5–P8, PO3, PO4, PO7, and PO8, were averaged for the time window from 125 to 145 ms based on the root mean square and the local maximum around Oz.

To ensure a good signal‐to‐noise ratio, we calculated the standardized measurement error for the mean amplitude of each selected electrode cluster within the time window of interest (Luck et al. 2021). The standardized measurement error gauges the precision of an individual's ERP amplitude by estimating the standard error of the mean from single trials prior to averaging. Instead of removing participants using a strict trial‐count cutoff, we excluded those with high measurement noise—specifically, individuals whose standardized measurement error exceeded two standard deviations above the group mean in at least one condition. This method ensures that the results are not influenced by trial‐to‐trial variability, even when the number of trials is limited. Consequently, the analysis of the frontal electrode clusters was based on 31 datasets, the analysis of the parietal electrode clusters on 30 datasets, and the analysis of the P1 on 32 datasets. For the P1‐based regression, the datasets used were the same ones included in the analysis of the respective SME subcomponent.

#### 2.4.3 Frequency Responses

Because memory processes can exhibit a latency jitter across trials, they are not strictly phase‐locked to stimulus onset and give rise to so‐called induced oscillatory activity. To capture these non‐phase‐locked dynamics, spectral changes in oscillatory activity were analyzed using single‐trial Morlet wavelet analysis of individual experimental epochs. Time‐domain averaging would attenuate these effects by obscuring trial‐by‐trial variability (see, e.g., Gruber et al. 2004, 2008; Tallon‐Baudry and Bertrand 1999). Moreover, the evoked response (i.e., the ERP) was subtracted from each trial before performing the frequency decomposition to enable analysis of the non‐phase‐locked components of the signal (for details, see, e.g., Busch et al. 2006). Thus, 60 wavelets from 1 to 30 Hz were computed with a frequency resolution of 0.5 Hz. The procedure provides a time‐by‐frequency (TF) representation of the data by yielding a time‐varying magnitude of the signal for each frequency band. The number of trials per condition was accounted for and matched across conditions (*M* = 39.5 ± 12.8 SD). Participants with fewer than 20 trials per condition after matching were excluded from analysis of the spectral changes. Consequently, the analyses of the frequency responses were based on 28 datasets. The relevant time window and electrodes of interest were derived from previous literature. The theta‐band response has been reported to reliably differentiate between subsequently remembered and forgotten stimuli from 300 to 1000 ms after stimulus onset (Osipova et al. 2006), with a narrower time window for frontal effects during pictorial encoding (i.e., 400–800 ms; White et al. 2013). Consequently, a time‐frequency (TF)‐plot, depicting the magnitude of the data per individual frequency band varying over time, was calculated for frontal electrodes (Fz, FCz, see Figure 3A), against which the frequency range and time window were counterchecked. This approach resulted in the selection of the frequency range of 3–6 Hz and the time window of 400–700 ms after stimulus onset. The topography of the grand mean across all conditions and the respective time window was used to countercheck which frontal electrodes around Fz to average for analyses, resulting in electrodes AFz, AF3, and AF4. The same procedure was applied to the alpha‐band response. Alpha‐band activity during attentional object encoding has predominantly been observed 300–900 ms after stimulus onset over posterior/occipital–parietal electrode sites (Johnsdorf et al. 2026; Kisker, Johnsdorf, et al. 2025a; van Diepen et al. 2016; see also Klimesch, Doppelmayr, Pachinger, and Ripper 1997; Klimesch, Doppelmayr, Schimke, and Ripper 1997; Clayton et al. 2018). The same approach as for the theta‐band response was applied, resulting in the frequency range of 10–13 Hz, the time window from 400 to 900 ms after stimulus onset, and electrodes Oz, POz, O1, O2, PO3, PO4, PO7, PO8, and P5–P8.

### 2.5 Statistical Analyses

As the general approach, inferential statistics were conducted, and effect sizes, partial eta squared (*η* ^*2*^), and Cohen's *d* were reported. If applicable, post hoc *t*‐tests were conducted and corrected for multiple comparisons using the false discovery rate (FDR) according to the Benjamini–Hochberg method (Benjamini and Hochberg 1995). The inferential approach was complemented by Bayesian statistics. JASP (JASP Team 2024; Kelter 2020) was used to calculate the *BF*_*10*_. A *BF*_*10*_ > 1 favors the H1, while a *BF*_*10*_ < 1 favors the H0. The following thresholds were applied as recommended for JASP (Kelter 2020): *BF*_*10*_ < 1—anecdotal evidence for H0, *BF*_*10*_ < 0.33—moderate evidence for H0, *BF*_*10*_ < 0.01—strong evidence for H0, *BF*_*10*_ < 0.03—very strong evidence for H0; *BF*_*10*_ > 1—anecdotal evidence for H1, *BF*_*10*_ > 3—moderate evidence for H1, *BF*_*10*_ > 10—strong evidence for H1, *BF*_*10*_ > 100—very strong evidence for H1.

#### 2.5.1 Behavioral Data

Participants' performance on the encoding task was calculated separately for the VR and PC encoding conditions. Encoding task performance was quantified as the proportion of correct responses relative to all responses in the encoding task. It was compared between encoding conditions using a two‐tailed paired‐sample *t*‐test. Responses to the retrieval task were used to classify stimuli as remembered or forgotten during the encoding phase. In the course of the revision process, an exploratory analysis of the sensitivity index *d*‐prime (*d'*) was added using a two‐tailed one‐sample *t*‐test to compare signal detection depending on VR‐based and PC‐based encoding. D′ quantifies the participant's ability to distinguish between previously seen items and novel distractors (*d'* = *z (hits)—z (false positive responses)*; Swets et al. 1961). Further analyses of the retrieval phase, in particular analyses of potential interaction effects between encoding and retrieval modality on memory performance, will be presented in a forthcoming companion publication.

#### 2.5.2 Electrophysiological Data

To analyze the SMEs, a 2 × 2 rmANOVA with the within‐subjects factor *Presentation Mode* (VR, PC) and *Subsequent Memory* (remembered, forgotten) was applied for each electrophysiological measure, i.e., for three ERP components, the theta‐band response, and the alpha‐band response. If the rmANOVA indicated a significant interaction between the two factors, post hoc paired‐samples *t*‐tests were conducted for further comparisons. Significant main effects of factors with two levels were not followed by post hoc *t*‐tests, as these comparisons are statistically equivalent to the corresponding *F*‐tests.

To ensure that potential modality‐specific variations in early visual processing (e.g., variations in perceived stimulus size) did not predict downstream encoding effects, we compared P1 amplitudes across conditions. A 2 × 2 rmANOVA with the within‐subjects factor *Presentation Mode* (VR, PC) and *Subsequent Memory* (remembered, forgotten) was applied. Additionally, we conducted linear regressions using the difference in mean P1 amplitude between encoding conditions as a predictor. To this end, a difference score of the encoding media was calculated (ΔEncodingMedium = mean (VR‐Remembered, VR‐Forgotten)—mean (PC‐Remembered, PC‐Forgotten)) for each target ERP component as well as for the frequency responses. These media difference scores were regressed on the corresponding difference score of the P1 amplitude (ΔP1 = mean P1(VR‐Remembered, VR‐Forgotten)—mean P1(PC‐Remembered, PC‐Forgotten)), resulting in the regression formula: Δ EncodingMedium_i_ = *β*_*0*_ + *β*_*1*_ * ΔP1_i_ + *ε*_*i*_; where *β*_*0*_ represents the intercept, *β*_*1*_ is the regression coefficient for the P1 difference predictor, and *ε* denotes the residual error term for subject *i*.

## 3 Results

### 3.1 Behavioral Data

Participants' hit rate in the encoding task was approximately 84% across *Presentation Mode*s, i.e., approximately 167 objects per condition were correctly categorized according to their size (*t*(35) = −1.41, *p* = 0.167, *M*_VR_ = 165.89, *d* = −0.24; *BF*_*10*_ = 0.45; SE_VR_ = 3.77, *M*_PC_ = 168.47, SE_PC_ = 3.40). Moreover, they subsequently remembered approximately 76% of the encoded stimuli independent of *Presentation Mode*, i.e., approximately 152 objects per condition (*t*(35) = 0.699, *p* = 0.489, *d* = 0.12; *BF*_*10*_ = 0.23; *M*_VR_ = 76.71%, SE_VR_ = 1.98%, *M*_PC_ = 75.40%, SE_PC_ = 11.26%). In congruence, *d'* indicated good ability to discriminate between old and new stimuli independent of *Presentation Mode* (*t*(35) = 0.90, *p* = 0.377, *d* = 0.07; *BF*_*10*_ = 0.25; *M*_VR_ = 0.80, SE_VR_ = 0.04, *M*_PC_ = 0.79, SE_PC_ = 0.03).

### 3.2 Event‐Related Potentials

#### 3.2.1 ERP Effect at Frontal Sensors

**FIGURE 2:**
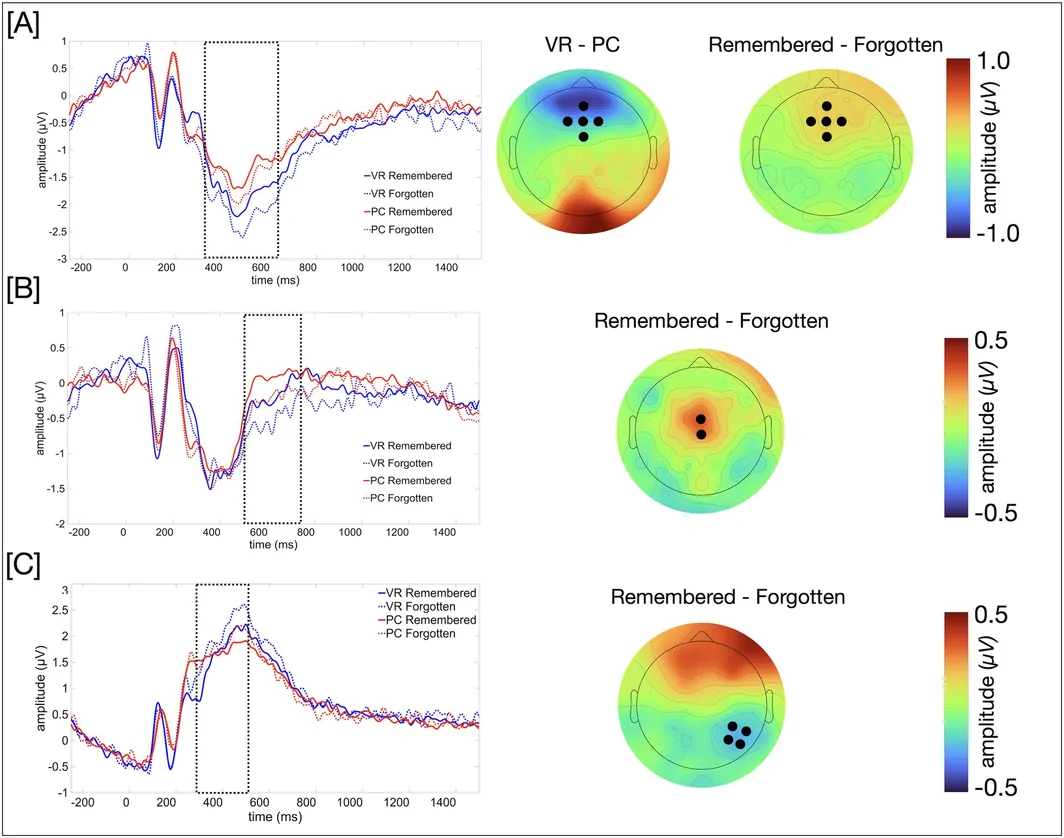
Illustration of the event‐related response for the subsequent memory effects. *Note:* The black dots in the topographies indicate the electrodes averaged for statistical comparisons across conditions, and the dotted lines indicate the analyzed time windows. The topographies depict the significant main effects. The line plots depict the amplitude across time averaged across (A) electrodes Fz, FCz, AFz, F1, and F2; (B) electrodes FCz and Cz, and (C) electrodes P4, P6, CP4, and CP6.

Significant main effects of *Presentation Mode* (*F*(1, 30) = 4.61, *p* = 0.040, *η* ^*2*^ = 0.13) and *Subsequent Memory* were observed (*F*(1, 30) = 4.40, *p* = 0.045, *η* ^*2*^ = 0.13), whereas their interaction did not reach significance (*F*(1, 30) = 0.60, *p* = 0.444, *η* ^*2*^ = 0.02). Independent of *Subsequent Memory*, objects encoded in VR elicited a more negative response compared to PC‐objects. Moreover, objects subsequently remembered elicited a less negative response than those subsequently forgotten (Figure 2A). The Bayesian ANOVA yielded anecdotal evidence for Presentation Mode (*BF*_*incl*_ = 2.29) and Subsequent Memory (*BF*_*incl*_ = 1.08), and moderate evidence against the Presentation Mode × Subsequent Memory interaction (*BF*_*incl*_ = 0.26).

#### 3.2.2 ERP Effect at Centrofrontal Sensors

Again, a significant main effect of *Subsequent Memory* (*F*(1, 30) = 12.57, *p* = 0.001, *η* ^*2*^ = 0.30) indicated that subsequently remembered objects elicited a less negative response than subsequently forgotten objects. However, no main effect of *Presentation Mode* (*F*(1, 30) = 2.04, *p* = 0.163, *η* ^*2*^ = 0.06) and no interaction of both factors (*F*(1, 30) = 0.05 *p* = 0.817, *η* ^*2*^ > 0.01) were observed (Figure 2B). Congruently, the Bayesian ANOVA yielded strong evidence for Subsequent Memory (*BF*_*incl*_ = 13.91), but anecdotal evidence against Presentation Mode (*BF*_*incl*_ = 0.79), and moderate evidence against the Presentation Mode × Subsequent Memory interaction (*BF*_*incl*_ = 0.27).

#### 3.2.3 ERP Effect at Parietal Sensors

A significant main effect of *Subsequent Memory* (*F*(1, 30) = 6.33, *p* = 0.018, *η* ^*2*^ = 0.18) indicated that subsequently remembered objects elicited a less positive response than subsequently forgotten objects. No main effect of *Presentation Mode* (*F*(1, 30) = 0.81, *p* = 0.375, *η* ^*2*^ = 0.03) and no interaction of both factors (*F*(1, 30) = 0.51, *p* = 0.482, *η* ^*2*^ = 0.2) were observed (Figure 2C). In congruence, the Bayesian ANOVA yielded anecdotal evidence for Subsequent Memory (*BF*_*incl*_ = 2.40) but anecdotal evidence against Presentation Mode (*BF*_*incl*_ = 0.59) and moderate evidence against the Presentation Mode × Subsequent Memory interaction (*BF*_*incl*_ = 0.33).

#### 3.2.4 P1

No significant differences were found across conditions in the P1 amplitude (all *F*s(1, 31) < 3.10, all *p*s > 0.09, all *η* ^*2*^ < 0.09). The Bayesian ANOVA was in congruence with these findings (all *BF*_*incl*_ [0.29–0.77]). Moreover, none of the differences between both ERP and frequency band responses to VR and PC conditions were significantly predicted by the difference in their P1 amplitudes (all *R* ^2^ ≤ 0.16, *β*_*1*_ range: 0.08–0.28, all *t*s ≤ 1.71, all *p*s ≥ 0.100). The corresponding Bayesian regressions indicated anecdotal evidence for the null hypothesis (all *BF*_*incl*_ [0.37–0.74]), except regarding the ERP at parietal sensors, for which anecdotal evidence for the alternative hypothesis was observed (*BF*_*incl*_ = 1.02).

### 3.3 Frequency Responses

#### 3.3.1 Theta‐Band Response

**FIGURE 3:**
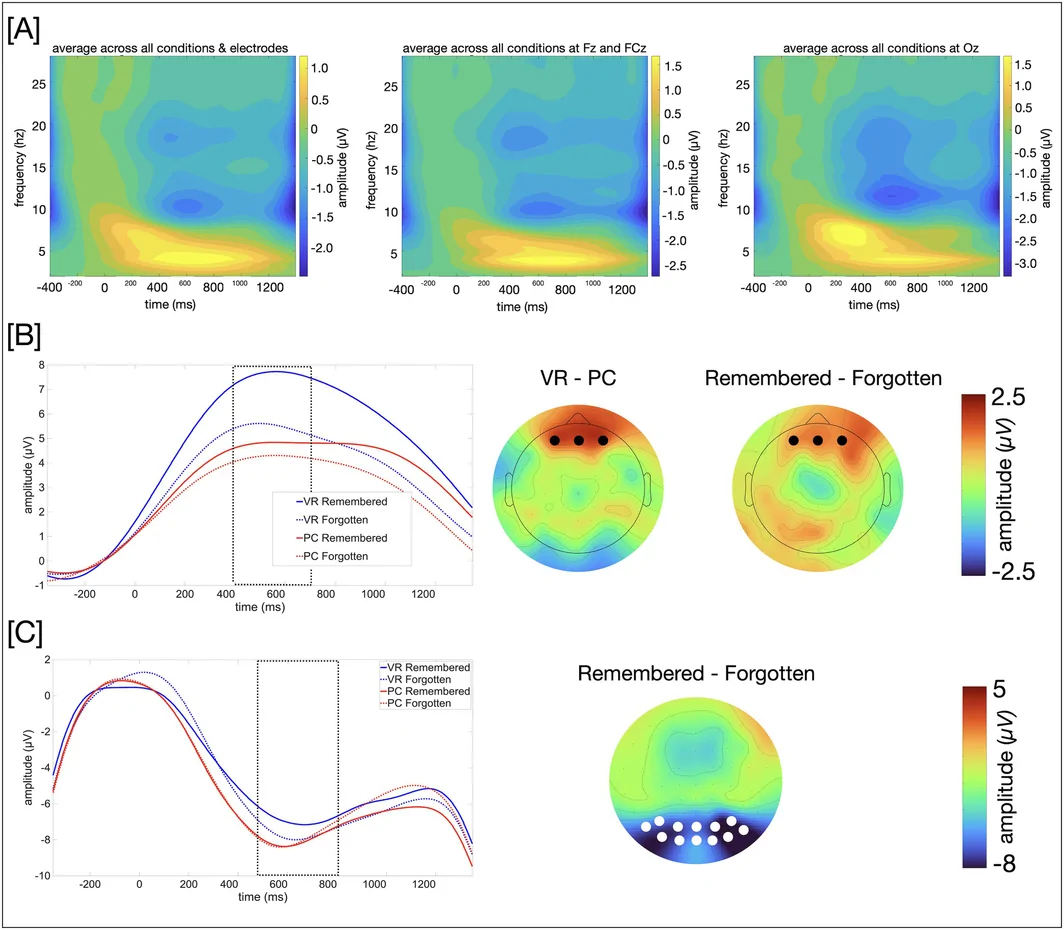
Time‐frequency plots, topographical amplitude distribution, and line plots of the frequency responses. *Note:* Panel (A) depicts the time‐frequency plots averaged across all, frontal (Fz, FCz), and occipital (Oz) electrodes. Panel (B) depicts the line plot and topographical amplitude distribution for the theta‐band response, whereas Panel (C) depicts the line plot and topographical amplitude distribution for the alpha‐band response. The topographies depict the significant main effects. The dotted lines in the line plots indicate the time window averaged for analysis, and the dots on the topographies indicate the electrodes averaged for analysis (Theta‐band response: AFz, AF3, and AF4; Alpha‐band response: Oz, POz, O1, O2, PO3, PO4, PO7, PO8, and P5–P8).

The 2 × 2 rmANOVA yielded significant main effects of *Presentation Mode* (*F*(1, 27) = 4.32, *p* = 0.047, *η* ^*2*^ = 0.13) and *Subsequent Memory* (*F*(1, 27) = 4.82, *p* = 0.037, *η* ^*2*^ = 0.15), whereas their interaction did not reach significance (*F*(1, 30) = 2.08, *p* = 0.161, *η* ^*2*^ = 0.07). Independent of *Subsequent Memory*, objects encoded in VR elicited a more positive theta‐band response compared with objects encoded on a PC. Moreover, objects subsequently remembered elicited a more positive response than those subsequently forgotten (Figure 3B). These results were reflected by the Bayesian ANOVA, which indicated anecdotal evidence for Subsequent Memory (*BF*_*incl*_ = 1.17) and Presentation Mode (*BF*_*incl*_ = 1.75) but anecdotal evidence against the Presentation Mode × Subsequent Memory interaction (*BF*_*incl*_ = 0.79).

#### 3.3.2 | Alpha Band Response

No significant differences were found between conditions in the alpha‐band response (all *F*s(1, 27) < 1, all *p*s > 0.59, all *η* ^*2*^ < 0.12; Figure 3C). The Bayesian ANOVA was congruent with these findings (all *BF*_*incl*_ [0.29–0.32]), providing moderate evidence for the null hypothesis.

## 4 Discussion

The present study investigated whether successful memory formation in immersive VR and PC conditions relies on comparable or modality‐specific configurations of encoding processes, including perceptual‐attentional, contextual, and elaborative processes. To this end, participants incidentally encoded everyday objects either in immersive VR or in a matched PC condition while EEG was recorded, and then completed an unannounced recognition test. Using a subsequent memory approach, we compared encoding‐related activity for subsequently remembered and subsequently forgotten objects within and across presentation modes. Across behavioral measures, encoding task performance and subsequent item memory did not differ between VR‐based and PC‐based encoding. At the electrophysiological level, subsequent memory effects (SMEs) were observed in the ERP at frontal and parietal electrode clusters, as well as in the theta‐band response at frontal sensors, indicating that subsequently remembered objects were processed differently during encoding than subsequently forgotten objects. These effects did not differ significantly between VR‐ and PC‐based encoding, providing no clear evidence that successful encoding was supported by modality‐specific process configurations. Nevertheless, presentation mode modulated overall encoding‐related activity, as the VR condition elicited a more negative early ERP response at frontal sensors and higher theta‐band amplitudes compared with the PC condition. Thus, the present findings suggest that immersive VR generally affects object processing during encoding, but do not provide clear evidence for modality‐specific mechanisms underlying successful memory in the current design.

### 4.1 Comparable Encoding Processes Across Presentation Modes

We hypothesized that encoding in VR might facilitate perceptual‐attentional, contextual, and elaborative processing, which would be reflected in relatively stronger SMEs in the theta‐band response and the family of ERPs linked to successful encoding. In contrast, the analyzed markers suggest that the successful encoding of everyday objects relied on comparable processes and their magnitudes across both presentation modes. Although VR altered overall neural responses during encoding, presentation mode did not modulate the responses that differentiated subsequently remembered from subsequently forgotten objects. This pattern was evident both behaviorally and in the ERP data: subsequent item memory did not differ between VR and PC, and the SMEs observed at frontal and parietal electrode clusters emerged independently of presentation mode in all time windows. Functionally, these SMEs suggest that successful item encoding relied on a shared configuration of object‐centered processes across both modalities. The early SME observed at frontal sensors may reflect semantic or task‐relevant conceptual processing, the later SME observed at frontal sensors sustained conceptual or elaborative processing, and the SME observed at parietal sensors item‐specific processing or item‐context binding (Kamp et al. 2017; Mecklinger and Kamp 2023). Such processes were likely promoted by the size‐judgment task, which required object identification and access to real‐world size knowledge in both conditions.

These findings are theoretically informative because the present VR–PC comparison was designed to minimize differences unrelated to the presentation medium. Both conditions were based on the same virtual environment, with object identity, task demands, and the design of the environment closely matched. The conditions therefore primarily differed in features inherent to the respective presentation modes, including stereoscopic depth cues and egocentric spatial information available in immersive VR. The task of estimating the object's real‐life size relative to a shoebox afforded semantic processing, size perception, and possibly the maintenance of a mental object representation (see Naspi et al. 2021). Across many studies, instructions that require meaning‐based or conceptually rich processing generally yield larger, more reliable gains in episodic memory than simple perceptual tasks (Craik and Tulving 1975; Peng et al. 2024). For example, Chen et al. (2025) demonstrated that the contribution of conceptual information to memory is greater than that of perceptual information when the encoding task targets the former. The shared task demands may thus have been more crucial for successful encoding than the modality‐specific affordances isolated in the present design. However, visual detail also predicts successful recognition and helps to avoid false alarms (Naspi et al. 2021). The stereoscopic depth cues provided by VR might thus generally facilitate the evaluation of object size. However, since the real‐life size had to be estimated, the evaluation afforded retrieval of factual knowledge about the object. Given that previously gained factual knowledge is available across presentation modes, the stereoscopic cues might provide less benefit for the task than expected. In this sense, the present results suggest that stereoscopic depth cues, egocentric perspective, and spatial embedding may alter how objects are processed, but do not necessarily change the processes that determine whether an object is later recognized, at least if the task does not directly rely on that information. In such task configurations, targeting conceptual information during encoding might even limit the processing of perceptual information not directly linked to the task (Chen et al. 2025).

A related explanation can be derived from the distinctiveness account discussed by Mecklinger and Kamp (2023). According to this account, distinctive features of an encoded stimulus or event contribute to subsequent memory effects primarily when the distinctive feature is integral to the encoded item and relevant for later retrieval. Applied to the present study, the stereoscopic and contextual features of VR may not have been perceived as being integral to the object representation required for later item recognition. Consequently, VR‐specific features could modulate general encoding‐related activity without producing modality‐specific subsequent memory effects.

Alternatively, the incidental encoding task may also have contributed to the absence of modality‐specific SMEs. ERP SMEs are known to depend on task characteristics and on the extent to which participants engage in strategic or elaborative encoding processes (Mecklinger and Kamp 2023). Particularly, SMEs have been reported to be diminished or even absent under incidental encoding conditions (Cycowicz and Friedman 1999). In contrast, the neural correlates linked to encoding were sensitive to subsequent memory across presentation modes in the current study. However, incidental encoding may have limited the extent of the effects and their potential interactions. Because encoding was incidental, participants may not have strategically used the additional spatial and contextual information available in VR. Thus, VR‐specific features such as stereoscopic depth cues or spatial context may have affected general processing during encoding, but may not have been strategically used to support later item recognition.

Overall, the analyzed markers of successful encoding were sensitive to subsequent memory independent of the two presentation modes, suggesting that encoding relies on consistent processes of comparable magnitude across both modes. However, the results do not imply that VR‐specific affordances are irrelevant for successful encoding under all conditions. Rather, the present findings suggest that the specific characteristics examined in an otherwise matched environment did not substantially alter the processes linked to successful subsequent memory. This provides a foundation for future studies that systematically vary additional features of immersive experience, such as contextual richness, embodied exploration, or active interaction.

### 4.2 Modality‐Specific Differences During Encoding

Although the encoding processes associated with subsequent memory did not differ across presentation modes, VR potentially modulated overall encoding‐related object processing. The theta‐band response was enhanced both for VR relative to PC and for subsequently remembered relative to subsequently forgotten objects, but these effects did not interact. The present pattern may therefore indicate that the theta‐band response captured both a perception‐related component linked to object processing and a modality‐independent component of successful item encoding. The stronger theta‐band response to the VR condition, independent of subsequent memory, could indicate enhanced visuospatial processing (Tang et al. 2022), specifically contextual processing by means of the integration of stereoscopic cues (Kisker, Johnsdorf, et al. 2025b), or cognitive load (Dan and Reiner 2017; Slobounov et al. 2015). Studies comparing different modes of presentation commonly report higher theta‐band responses for VR than for PC stimuli across passive viewing and active tasks, and are often interpreted as reflecting enhanced processing of immersive or three‐dimensional material (Li et al. 2020; Malik et al. 2015; Slobounov et al. 2015; Tang et al. 2022; Xu and Sui 2021). However, findings based on the theta‐band response are not unambiguous: findings linking it to cognitive load suggest that 3D presentation may instead reduce cognitive load relative to 2D conditions (Dan and Reiner 2017; Kisker, Johnsdorf, et al. 2025b; Sagehorn et al. 2024). Thus, modulations of theta‐band activity in response to VR may reflect multiple rather than a single mechanism. Given that the present design closely matched task demands and visual context across VR and PC while retaining presentation‐mode‐specific differences, the VR‐related increase in the theta‐band response may reflect enhanced visuospatial integration of object and visual context. However, because the theta‐band response has been linked to multiple processes, including spatial processing, cognitive control, working memory, and cognitive load, this interpretation should be approached with caution. Critically, the absence of a significant interaction suggests that VR increased the theta‐band response in general but did not clearly amplify the theta‐related processes that distinguished subsequently remembered from subsequently forgotten objects.

A related interpretation may apply to the early SME observed at frontal sensors. Although frontal ERP effects in this time range have been associated with semantic or conceptual processing in the context of subsequent memory effects (Mecklinger and Kamp 2023), the more negative response to VR‐based than to PC‐based presentation should not be equated with successful semantic encoding. It occurred independently of later memory and, therefore, more likely reflects general differences in task‐relevant object evaluation. As aforementioned, the encoding task required object identification, access to factual knowledge, and their integration under both conditions. Under VR conditions, this evaluation may have additionally involved integrating stereoscopic depth cues, egocentric perspective, and the spatial context of the virtual scene. Thus, in line with the theta‐band response, the early ERP difference at frontal sensors may indicate that evaluating objects according to the encoding task was supported by partly distinct processing demands across presentation modes, without selectively modulating the processes that predicted subsequent memory.

Importantly, these effects can neither exclusively be attributed to differences in early visual processing nor to attentional effects. Neither did the P1 differentiate between conditions depending on the presentation mode and subsequent memory, nor did the P1‐difference score of presentation modes predict subsequent effects. Similarly, neither the ERP response at the parietal electrode cluster associated with attention allocation nor the alpha‐band response associated with attentional processing differed between the VR and PC conditions. Together, these findings indicate that differences between both modes of presentation during encoding are more likely to reflect post‐perceptual object evaluation or visuospatial integration than basic visual or attentional differences alone.

## 5 Conclusion

The present study examined whether successful object encoding in immersive VR and PC conditions relies on comparable or modality‐specific encoding processes. Using ERP and theta‐band subsequent memory effects, we found that later remembered objects differed from later forgotten ones during encoding, but that these effects were not significantly modulated by the mode of presentation. At the same time, VR elicited higher theta‐band responses and a more negative ERP response at frontal sensors, indicating that immersive presentation altered general object processing during encoding. Thus, immersive VR changed the conditions under which objects were processed, but did not provide clear evidence for modality‐specific mechanisms of successful encoding in the present task. This pattern may reflect the closely matched object material, task demands, and visual environment, as well as the fact that VR‐specific features such as stereoscopic depth, egocentric perspective, and spatial embedding were not necessarily required for later item recognition. The present study, therefore, provides a controlled baseline for future work examining which additional affordances of immersive experience shape successful memory formation.

## 6 Limitations

Successful encoding was operationalized by subsequent item recognition. Although this approach is well‐established and allowed us to examine whether encoding activity predicted subsequent item memory, it may be less sensitive to VR‐specific effects on contextual or source‐related aspects of memory. Modality‐specific encoding effects may become more apparent when subsequent memory is defined by source accuracy, recollective experience, confidence, or the retrieval of contextual details (Kisker et al. 2026; Kisker, Soethe, et al. 2025).

Moreover, previous ERP studies on subsequent memory effects have commonly used mastoid references (e.g., Forester and Kamp 2023; Kamp et al. 2017). In contrast, the average reference was selected in the present study for comparability with previous joint VR‐EEG studies (Johnsdorf et al. 2023; Lange et al. 2025) and its common application in mobile EEG preprocessing (Klug et al. 2022; Klug and Gramann 2021), i.e., preprocessing of conditions more susceptible to movement‐related artifacts. To assess the robustness of the results to this choice, we additionally conducted exploratory repeated‐measures ANOVAs including the reference as a factor for each ERP component (i.e., average reference versus TP9 and TP10 as mastoid references). These analyses indicated significant effects related to *Reference* only for the ERP component at central electrodes in the 550–750 ms time window. Repeating the original analysis with mastoid‐referenced data of this component resulted in an additional main effect of *Presentation Mode*. Details are provided in Data S1.

The absence of significant interaction between the mode of presentation and subsequent memory performance should not be interpreted as evidence for equivalence between VR and PC. This is particularly relevant for the theta‐band response, where the interaction did not reach significance, showed a small‐to‐moderate effect size, and the Bayes factor provided only anecdotal evidence for the null hypothesis. Although the effect size provides no evidence for modality‐specific theta SMEs, it suggests that future studies may benefit from designs with greater sensitivity to such interactions. Future studies with larger samples and designs optimized for subsequent memory contrasts, e. g., including intentional encoding, may provide a more sensitive test of modality‐specific encoding processes.

Although the VR and PC conditions were closely matched regarding object identity, task demands, and visual context, presentation‐mode‐specific features necessarily remained confounded. In particular, the present design does not allow the effects of stereoscopic depth cues to be separated from other features associated with immersive HMD presentation. Thus, the observed differences should be interpreted as effects of the presentation mode as implemented here rather than as effects of immersion per se. Future studies could systematically manipulate individual features, for example by including stereoscopic PC or monoscopic VR stimuli.

Last but not least, the retrieval procedures were adapted from two previous studies (Kisker et al. 2026; Kisker, Soethe, et al. 2025) and therefore differed slightly in the visual arrangement of the confidence‐rating display and the duration of the inter‐trial interval to faithfully replicate the original VR and PC retrieval paradigms. The modified confidence‐rating layout in VR was required by the restricted field of view of the HMD, whereas the longer ITI reflected technical requirements of the original VR retrieval implementation. However, these differences are unlikely to have influenced the present findings, as each retrieval trial was preceded by a jittered fixation period and the electrophysiological analyses reported here were based exclusively on stimulus‐locked encoding activity.

## Author Contributions

**Joanna Kisker:** conceptualization, methodology, software, investigation, formal analysis, project administration, visualization, writing – review and editing, writing – original draft. **Merle Sagehorn:** writing – review and editing. **Thomas Gruber:** conceptualization, methodology, supervision, resources, writing – review and editing.

## Funding

The authors have nothing to report.

## Ethics Statement

The studies involving human participants were reviewed and approved by the local ethics committee of Osnabrück University, Germany (reference: Ethik‐59/2025).

## Consent

All participants gave their informed consent to participate in the study and for the publication of their anonymized data.

## Conflicts of Interest

The authors declare no conflicts of interest.

## Supporting information


**Table S1:1** Results of exploratory repeated‐measures ANOVAs including EEG Reference as an additional within‐subject factor.
**Table S1:2** Exploratory analysis of the 550–750 ms ERP component using mastoid‐referenced data.

## Data Availability

The datasets presented in this study are available in the online repository OSF: https://osf.io/yzfpx/overview?view_only=7bc737194c4a48bab36aef9288491c8b. Software applications and custom code can be obtained from the corresponding author upon reasonable request.
