# Supplementary material for: Electrophysiological Correlates of Encoding Indicate Altered Processing but Comparable Subsequent Memory Effects Across Virtual Reality and Desktop‐Based Conditions
Source: Eur J Neurosci. 2026 Aug 27;64(5):e70664. doi: 10.1111/ejn.70664 (PMC13522653; doi:10.1111/ejn.70664)
Supplement: Supplementary file 1 [file EJN-64-0-s001.docx]

**Supplementary Material:** *Electrophysiological Correlates of Encoding Indicate Altered Processing but Comparable Subsequent Memory Effects Across Virtual Reality and PC Conditions*

Joanna Kisker*, Merle Sagehorn, Thomas Gruber

*S1. Robustness Analyses of EEG Reference Choice*

To evaluate whether the choice of EEG reference influenced the reported ERP findings, exploratory repeated-measures ANOVAs including Reference as an additional within-subject factor were conducted for each ERP component. Table S1.1 summarizes only the effects involving Reference. No interactions involving Reference and Subsequent Memory were observed for any ERP component. Because significant reference-related effects were observed for the 550–750 ms ERP component only, the original 2 × 2 repeated-measures ANOVA was repeated using mastoid-referenced data for this component (Table S1.2). Re-analysis of this component using mastoid-referenced data yielded an additional main effect of Presentation Mode, which does not reach significance for the data re-referenced to average reference. The analyses based on the average reference were thus maintained in correspondence with previous joint VR-EEG studies (Johnsdorf et al., 2023; Lange et al., 2025) and its common application in mobile EEG preprocessing (Klug et al., 2022; Klug & Gramann, 2021),

Table S1.1

*Results of exploratory repeated-measures ANOVAs including EEG Reference as an additional within-subject factor.*

| *Effect* | *350–650 ms*  *frontal ROI^a^* | *300–500 ms*  *parietal ROI^b^* | *550–750 ms*  *frontocentral ROI^c^* |
| --- | --- | --- | --- |
| *Reference* | *F*(1,30) = 1.79,  *p* = .192, η²ₚ = .056 | *F*(1,29) = 0.25,  *p* = .624, η²ₚ = .008 | *F*(1,30) = 6.60, *p* = .015, η²ₚ = .180 |
| *Reference × Presentation Mode* | *F*(1,30) = 2.26,  *p* = .143, η²ₚ = .070 | *F*(1,29) = 2.05,  *p* = .163, η²ₚ = .066 | *F*(1,30) = 5.64,  *p* = .024, η²ₚ = .158 |
| *Reference × Memory State* | *F*(1,30) = 0.03,  *p* = .859, η²ₚ = .001 | *F*(1,29) = 0.72,  *p* = .404, η²ₚ = .024 | *F*(1,30) = 0.43,  *p* = .516, η²ₚ = .014 |
| *Reference × Presentation Mode × Memory State* | *F*(1,30) = 0.51,  *p* = .481, η²ₚ = .017 | *F*(1,29) = 0.50,  *p* = .485, η²ₚ = .017 | *F*(1,30) = 2.88,  *p* = .100, η²ₚ = .087 |

*Note*. ^a^ Frontal ROI: averaged across electrodes Fz, FCz, AFz, F1, F2; ^b^ partietal ROI: averaged across electrodes P4, P6, CP4, CP6; ^c^ frontocentral ROI: averaged across electrodes Cz, FCz. ROI = Region of interest.

Table S1.2

*Exploratory analysis of the 550–750 ms ERP component using mastoid-referenced data.*

| ***Effect*** | ***Statistic*** |
| --- | --- |
| *Presentation Mode* | *F*(1,30) = 4.43, *p* = .044, η²ₚ = .129 |
| *Subsequent Memory* | *F*(1,30) = 4.79, *p* = .037, η²ₚ = .138 |
| *Presentation Mode × Subsequent Memory* | *F*(1,30) = 1.46, *p* = .236, η²ₚ = .046 |

*Note*. The results are consistent with those based on the data referenced to the average reference, except that the main effect of *Presentation Mode* reaches significance only for the mastoid-referenced data. Importantly, the main effect of *Subsequent Memory* is robust to the choice of reference.
